# Supplementary material for: Evidence supported by Mendelian randomization: impact on inflammatory factors in knee osteoarthritis
Source: Front Med (Lausanne). 2024 May 28;11:1382836. doi: 10.3389/fmed.2024.1382836 (PMC11165061; doi:10.3389/fmed.2024.1382836)
Supplement: Supplementary file 1 [file Data_Sheet_1.ZIP › data/GCST90274844_5e6/844.leaveoneout.pdf]

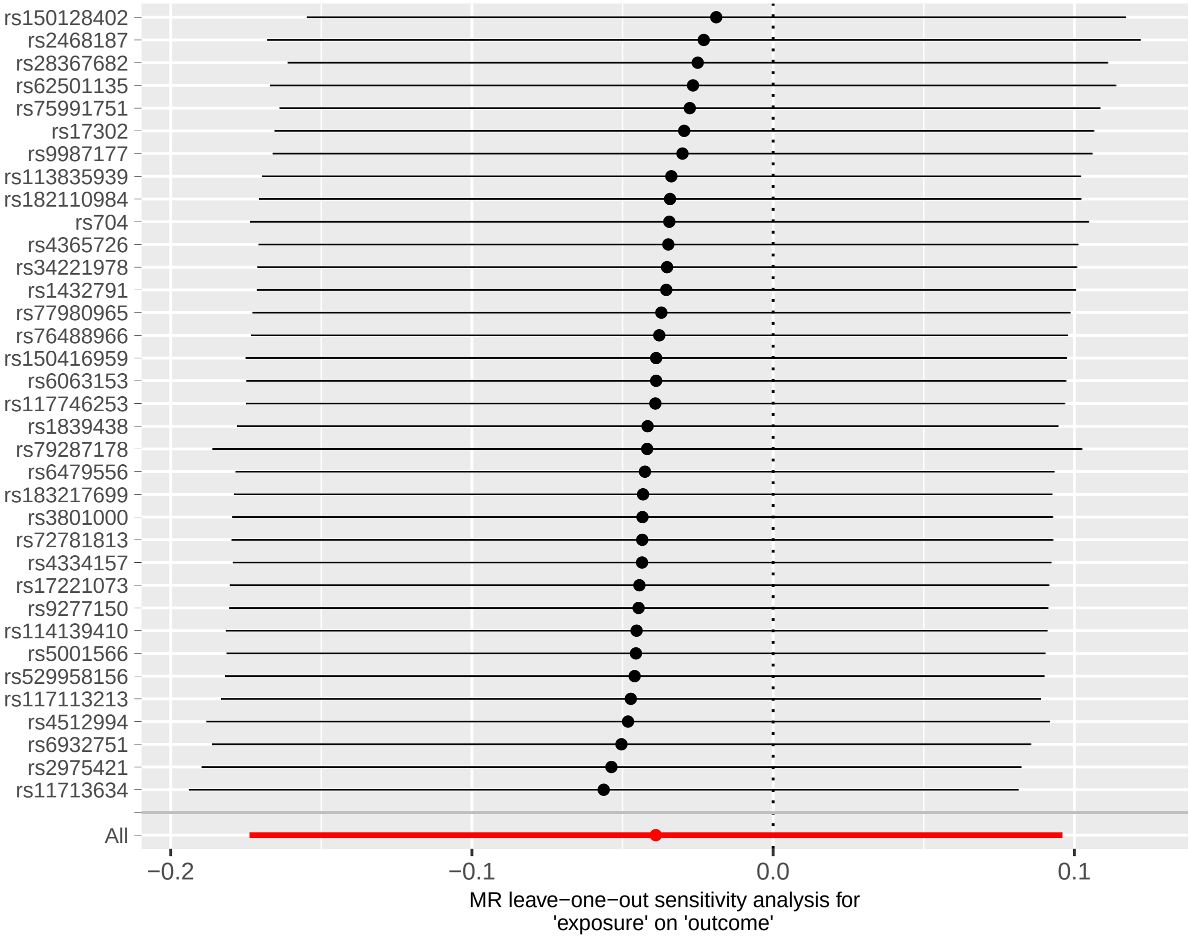
rs150128402
rs2468187
rs28367682
rs62501135
rs75991751
rs17302
rs9987177
rs113835939
rs182110984
rs704
rs4365726
rs34221978
rs1432791
rs77980965
rs76488966
rs150416959
rs6063153
rs117746253
rs1839438
rs79287178
rs6479556
rs183217699
rs3801000
rs72781813
rs4334157
rs17221073
rs9277150
rs114139410
rs5001566
rs529958156
rs117113213
rs4512994
rs6932751
rs2975421
rs11713634
All
−0.2
−0.1
0.0
0.1
MR leave−one−out sensitivity analysis for
'exposure' on 'outcome'
